# Histo-Blood Group Antigen Null Phenotypes Associated With a Decreased Risk of Clinical Rotavirus Vaccine Failure Among Children <2 Years of Age Participating in the Vaccine Impact on Diarrhea in Africa (VIDA) Study in Kenya, Mali, and the Gambia

**DOI:** 10.1093/cid/ciac910

**Published:** 2023-04-19

**Authors:** Lauren M Schwartz, Jennifer Oshinsky, Mardi Reymann, Mathew D Esona, Michael D Bowen, M Jahangir Hossain, Syed M A Zaman, Joquina Chiquita M Jones, Martin Antonio, Henry Badji, Golam Sarwar, Samba O Sow, Doh Sanogo, Adama Mamby Keita, Boubou Tamboura, Awa Traoré, Uma Onwuchekwa, Richard Omore, Jennifer R Verani, Alex O Awuor, John B Ochieng, Jane Juma, Billy Ogwel, Umesh D Parashar, Jacqueline E Tate, Irene N Kasumba, Sharon M Tennant, Kathleen M Neuzil, Ali Rowhani-Rahbar, M Elizabeth Halloran, Robert L Atmar, Marcela F Pasetti, Karen L Kotloff

**Affiliations:** Department of Epidemiology, School of Public Health, University of Washington, Seattle, Washington, USA; Vaccine and Infectious Diseases Division, Fred Hutchinson Cancer Research Center, Seattle, Washington, USA; Center for Vaccine Development and Global Health, University of Maryland School of Medicine, Baltimore, Maryland, USA; Department of Medicine, University of Maryland School of Medicine, Baltimore, Maryland, USA; Center for Vaccine Development and Global Health, University of Maryland School of Medicine, Baltimore, Maryland, USA; Department of Medicine, University of Maryland School of Medicine, Baltimore, Maryland, USA; Division of Viral Diseases, Centers for Disease Control and Prevention, Atlanta, Georgia, USA; Division of Viral Diseases, Centers for Disease Control and Prevention, Atlanta, Georgia, USA; Medical Research Council Unit, The Gambia at the London School of Hygiene & Tropical Medicine, Banjul, The Gambia; Medical Research Council Unit, The Gambia at the London School of Hygiene & Tropical Medicine, Banjul, The Gambia; Medical Research Council Unit, The Gambia at the London School of Hygiene & Tropical Medicine, Banjul, The Gambia; Medical Research Council Unit, The Gambia at the London School of Hygiene & Tropical Medicine, Banjul, The Gambia; Medical Research Council Unit, The Gambia at the London School of Hygiene & Tropical Medicine, Banjul, The Gambia; Medical Research Council Unit, The Gambia at the London School of Hygiene & Tropical Medicine, Banjul, The Gambia; Centre pour le Développement des Vaccins du Mali, Bamako, Mali; Centre pour le Développement des Vaccins du Mali, Bamako, Mali; Centre pour le Développement des Vaccins du Mali, Bamako, Mali; Centre pour le Développement des Vaccins du Mali, Bamako, Mali; Centre pour le Développement des Vaccins du Mali, Bamako, Mali; Centre pour le Développement des Vaccins du Mali, Bamako, Mali; Kenya Medical Research Institute, Center for Global Health Research, Kisumu, Kenya; Division of Global Health Protection, US Centers for Disease Control and Prevention, Nairobi, Kenya; Kenya Medical Research Institute, Center for Global Health Research, Kisumu, Kenya; Kenya Medical Research Institute, Center for Global Health Research, Kisumu, Kenya; Kenya Medical Research Institute, Center for Global Health Research, Kisumu, Kenya; Kenya Medical Research Institute, Center for Global Health Research, Kisumu, Kenya; Division of Viral Diseases, Centers for Disease Control and Prevention, Atlanta, Georgia, USA; Division of Viral Diseases, Centers for Disease Control and Prevention, Atlanta, Georgia, USA; Center for Vaccine Development and Global Health, University of Maryland School of Medicine, Baltimore, Maryland, USA; Department of Medicine, University of Maryland School of Medicine, Baltimore, Maryland, USA; Center for Vaccine Development and Global Health, University of Maryland School of Medicine, Baltimore, Maryland, USA; Department of Medicine, University of Maryland School of Medicine, Baltimore, Maryland, USA; Center for Vaccine Development and Global Health, University of Maryland School of Medicine, Baltimore, Maryland, USA; Department of Medicine, University of Maryland School of Medicine, Baltimore, Maryland, USA; Department of Epidemiology, School of Public Health, University of Washington, Seattle, Washington, USA; Department of Epidemiology, School of Public Health, University of Washington, Seattle, Washington, USA; Vaccine and Infectious Diseases Division, Fred Hutchinson Cancer Research Center, Seattle, Washington, USA; Department of Biostatistics, School of Public Health, University of Washington, Seattle, Washington, USA; Center for Inference and Dynamics of Infectious Diseases, Seattle, Washington, USA; Department of Medicine, Baylor College of Medicine, Houston, Texas, USA; Center for Vaccine Development and Global Health, University of Maryland School of Medicine, Baltimore, Maryland, USA; Department of Pediatrics, University of Maryland School of Medicine, Baltimore, Maryland, USA; Center for Vaccine Development and Global Health, University of Maryland School of Medicine, Baltimore, Maryland, USA; Department of Pediatrics, University of Maryland School of Medicine, Baltimore, Maryland, USA

**Keywords:** host genetics, HBGA phenotype, rotavirus vaccine failure

## Abstract

**Background:**

Previously studied risk factors for rotavirus vaccine failure have not fully explained reduced rotavirus vaccine effectiveness in low-income settings. We assessed the relationship between histo-blood group antigen (HBGA) phenotypes and clinical rotavirus vaccine failure among children <2 years of age participating in the Vaccine Impact on Diarrhea in Africa Study in 3 sub-Saharan African countries.

**Methods:**

Saliva was collected and tested for HBGA phenotype in children who received rotavirus vaccine. The association between secretor and Lewis phenotypes and rotavirus vaccine failure was examined overall and by infecting rotavirus genotype using conditional logistic regression in 218 rotavirus-positive cases with moderate-to-severe diarrhea and 297 matched healthy controls.

**Results:**

Both nonsecretor and Lewis-negative phenotypes (null phenotypes) were associated with decreased rotavirus vaccine failure across all sites (matched odds ratio, 0.30 [95% confidence interval: 0.16–0.56] or 0.39 [0.25–0.62], respectively]. A similar decrease in risk against rotavirus vaccine failure among null HBGA phenotypes was observed for cases with P[8] and P[4] infection and their matched controls. While we found no statistically significant association between null HBGA phenotypes and vaccine failure among P[6] infections, the matched odds ratio point estimate for Lewis-negative individuals was >4.

**Conclusions:**

Our study demonstrated a significant relationship between null HBGA phenotypes and decreased rotavirus vaccine failure in a population with P[8] as the most common infecting genotype. Further studies are needed in populations with a large burden of P[6] rotavirus diarrhea to understand the role of host genetics in reduced rotavirus vaccine effectiveness.

## Teaser

Assessing histo-blood group antigen (HBGA) phenotypes and rotavirus vaccine failure among children in sub-Saharan Africa, we found a significant relationship between null HBGA phenotypes and decreased rotavirus vaccine failure in a population with P[8] as the common infecting genotype.

In the last decade, following the introduction of a live-attenuated human monovalent vaccine (Rotarix; RV1) and a live-attenuated pentavalent human-bovine reassortant vaccine (RotaTeq; RV5), a significant reduction of rotavirus diarrhea morbidity and mortality rates was observed [1, 2]. Nonetheless, clinical trials and observational studies in sub-Saharan Africa and Asia have consistently estimated moderate rotavirus vaccine efficacy and effectiveness (40%–60%), compared with similar studies in high-resource settings (90%) [3–9]. Previously studied risk factors for rotavirus vaccine failure include high levels of maternal rotavirus antibodies during pregnancy and breastfeeding, concomitant oral polio vaccine administration, malnutrition, a high burden of coenteric pathogens, a greater force of infection, and a lack of diversity in the microbiome [10]. The role of genetically determined susceptibility to rotavirus has been proposed as a novel mechanism for rotavirus vaccine failure in low- and middle-income settings [11, 12].

Histo-blood group antigens (HBGAs) are expressed on the surface of many cell types, including intestinal epithelial cells, and are also detected as free molecules in the saliva, milk, and other biological fluids; they are thought to act as attachment factors for many enteric pathogens, including rotavirus [13]. Rotavirus is classified by typing of the 2 surface proteins: VP7 is a glycoprotein (G) and VP4 is a protease-sensitive protein (P) that is proteolytically cleaved into 2 smaller structural proteins, VP5 and VP8*. Rotavirus binds to sialo glycans or HBGAs on intestinal epithelial cells via VP8* in a genotype-specific manner [14, 15]. HBGAs are synthesized by sequential additions of monosaccharides encoded by 3 gene families: ABO, Lewis, and secretor. Genetic polymorphisms in secretor (*FUT2*) and Lewis (*FUT3*) gene families cause loss-of-function mutations, leading to null phenotypes (nonsecretor and Lewis negative). Expression of Lewis a, Lewis b, ABH antigens, and subsequent infectious disease susceptibility is dependent on the phenotypes of these gene families [16].

In vitro [17–19] and epidemiologic [20–28] studies have examined the relationship between HBGA phenotype and genotype-specific susceptibility to rotavirus diarrhea in several settings. Most studies have found nonsecretors to be at significantly decreased risk of P[8] rotavirus infections, with some also showing a decreased risk of P[4] infections. A low prevalence of Lewis-negative phenotype (<5%) and limited rotavirus genotypic diversity in high-income settings has made it challenging to study these relationships. Two studies showed an increased risk of P[6] rotavirus diarrhea in Lewis-negative participants [16, 22].

Based on this evidence, HBGA phenotype may be a risk factor for rotavirus vaccine failure in sub-Saharan Africa, where there is a greater diversity of circulating rotavirus genotypes [29–31] and null HBGA phenotypes are more common, especially the Lewis-negative phenotype [16]. Existing live oral rotavirus vaccines are effective by mimicking natural infection. Vaccine virus infects host cells in the gut epithelium and induces an immune response that will later target circulating genotypes of natural rotavirus infection [32].

While current rotavirus vaccines differ in their included G types (Rotarix: G1; RotaTeq: G1, G2, G3, G4), both include P[8] as the sole human rotavirus P component. Generally, both vaccines demonstrate cross-protection, with efficacy against heterotypic genotypes, that is, strains not included in the vaccine, at the same level as homotypic strains [33]. It has been hypothesized that individuals resistant to P[8] infections (nonsecretors and Lewis-negative children) may not be able to mount an immune response to existing vaccines and would be susceptible to non-P[8] wild-type infections. Several studies have assessed the interaction between HBGA phenotype and rotavirus vaccine seroconversion with varied results based on location and HBGA phenotype prevalence [22–25, 27]. Importantly, seroconversion is a surrogate, but not a correlate, of protection, and it remains unclear whether HBGA phenotype influences vaccine effectiveness, given its potential to affect both vaccine “take” and susceptibility to common wild-type rotavirus P types [34, 35].

The impact of HBGA phenotypes on rotavirus vaccine failure has been studied in limited settings, and even less so in regions of the world with the greatest burden of rotavirus diarrhea. We built on the existing infrastructure of the Vaccine Impact on Diarrhea in Africa (VIDA) study, a study assessing diarrheal diseases in The Gambia, Mali, and Kenya following rotavirus vaccine introduction, to explore the role of host genetics determinants in clinical rotavirus vaccine failure in vaccinated children 3–23 months of age.

## METHODS

### Participants and Study Design

The current study was nested within the VIDA study, a case-control evaluation of moderate-to-severe diarrhea (MSD) in The Gambia, Mali, and Kenya conducted between 2015 and 2018. The VIDA study assessed rotavirus vaccine effectiveness and population impact on the diarrheal burden, causes, and clinical consequence of MSD in children <5 years of age following rotavirus vaccine introduction. Participants were enrolled at 3 African study sites: Medical Research Council Unit The Gambia at the London School of Hygiene & Tropical Medicine [MRCG at LSHTM], Basse, The Gambia; Centre pour le Développement des Vaccins du Mali (CVD-Mali), Bamako, Mali; and the Centers for Disease Control and Prevention and Kenya Medical Research Institute [KEMRI], Siaya County, Kenya). All 3 countries introduced rotavirus vaccine in 2013 or 2014. Mali and The Gambia introduced RotaTeq, while Kenya introduced Rotarix. In April 2017, The Gambia switched to Rotarix.

Methods in VIDA are similar to those used in the Global Enteric Multicenter Study [36]. In brief, cases with a new episode (onset after ≥7 diarrhea-free days) of acute diarrhea (defined as ≥3 loose stools within 24 hours, for a period of <7 days) who were seeking care at sentinel healthcare centers serving a censused population at each site were evaluated for MSD. Those who had any of the following criteria were considered to have MSD: (1) sunken eyes, (2) loss of skin turgor, (3) intravenous hydration prescribed, (4) hospitalization recommended, or (5) dysentery. Cases were enrolled within 3 age groups (0–11, 12–23, or 24–59 months). Within each age group, we aimed to enroll 8–9 cases with MSD per fortnight, although during rotavirus season all cases with MSD seeking care were enrolled. Matched controls, without diarrhea for the 7 days before enrollment, were identified through the Health and Demographic Surveillance System at each site and enrolled at their homes. From 1 to 3 controls were matched to each case by residence (same or nearby village or community), sex, age group, and time (enrolled within 14 days of the patient’s enrollment date). This analysis includes only cases with MSD and matched controls 3–23 months of age.

### Data Collection and Procedures

After informed consent was obtained in the local language, the interviewer administered a standardized questionnaire to the parent/primary caretaker of cases and controls to collect epidemiologic and clinical data. Anthropometric measurements were also performed. Interviewers recorded the dates of all vaccines from a written document that included the vaccination card or, if a vaccination card was not available, from registers at the vaccine administration center. A single, fresh whole-stool sample was collected at enrollment from cases and matched controls and placed into cold storage within 1 hour of stool production [37].

For this study, saliva samples were collected from participants at the end of the interview using the SalivaBio Infant's Swab Method kit. Samples were collected ≥30 minutes after eating or breastfeeding; the child's mouth was washed with oral rehydration solution or clean water if breastfeeding had occurred during the previous 30 minutes. Saliva and stool samples were put on ice packs or refrigerated until taken to the site-specific laboratory. Saliva samples were frozen at −80°C during storage and shipment. If insufficient saliva was collected during the enrollment visit, additional saliva was collected during a 60-day follow-up visit.

### Rotavirus Infections and Genotyping

Detailed laboratory methods are available in the Supplementary Methods. Briefly, rotavirus VP6 antigen was detected at each site using the ProSpecT enzyme-linked immunosorbent assay (ELISA) rotavirus kit [38]. Among cases testing positive for rotavirus with ELISA, genotyping was performed with reverse-transcription polymerase chain reaction to determine the VP7 and VP4 genotypes, using conventional multiplexed 1-step amplification process with slight modifications [39]. Due to a change in laboratory site, genotyping for 38 cases from Mali was performed using methods different from those used in other cases (details in Supplementary Methods).

### Secretor and Lewis Phenotypes and Confirmatory Genotypes

The HBGA phenotype was determined by ELISA, testing for the presence of H-type 1, type A, type B, Lewis a, and Lewis b antigens in saliva (details in Supplementary Methods). Secretor status was defined as positive if type A, type B, Lewis b, or UEA-1 lectin assay (testing for H-type 1 antigen) results were positive. If all assay results were negative, the participant was considered a nonsecretor. Lewis status was defined as positive if Lewis a or Lewis b assay results were positive or as negative if both Lewis a and Lewis b results were negative. In a subset of saliva samples (42 included in the current analysis) secretor phenotype was confirmed with genetic sequencing. Genomic DNA was isolated from saliva cell pellets using a QIAamp DNA Blood Mini Kit. Sequence results were analyzed for the presence of single-nucleotide polymorphisms known to result in nonsecretor status.

### Statistical Analysis

The analysis was restricted to vaccinated rotavirus-positive cases with MSD 3–23 months of age and their vaccinated matched controls. Rotavirus-negative cases were not included in this analysis. Cases and matched controls included in this analysis had sufficient saliva to ascertain HBGA phenotype and documentation of ≥1 dose of rotavirus vaccine. Therefore, cases, defined as those in whom rotavirus diarrhea developed after a course of immunizations, are considered vaccine failures. Separate conditional logistic regression models were used to estimate the matched relative odds and associated 95% confidence intervals (CIs) for the association between rotavirus diarrhea and secretor status (secretor or nonsecretor) or Lewis status (Lewis-positive or Lewis-negative). The main analysis was stratified by infecting rotavirus genotype (P[8], P[6], or P[4]). Rotavirus-positive cases with MSD who had mixed or no genotyping results were excluded from these analyses, along with associated matched healthy controls.

Sensitivity analyses were conducted excluding participants with partial vaccination and/or stunting. These sensitivity analyses account for factors that might be independently associated with rotavirus diarrhea, which could introduce differences between cases and matched controls but are not associated with HBGA phenotype. Partially vaccinated was defined as 1 documented dose in Kenya and as 1 or 2 documented doses in Mali. In The Gambia, children were considered partially immunized if they received 1 or 2 doses and the first dose was given before 1 April 2017, when the Expanded Programme on Immunization administered RotaTeq. After this date, Rotarix was instituted programmatically and a child was considered partially immunized if 1 dose was provided. Stunting, an anthropometric measure that reflects chronic poor nutrition, recurrent infections or chronic diseases, was defined as height-for-age *z* score less than -2 standard deviations. Sensitivity analyses were not stratified by rotavirus genotype.

Analyses were performed using Stata software, version 16 (StataCorp), and R software, version 3.5. This study was approved by the ethical review committees at the University of Washington, Seattle; the University of Maryland, Baltimore; The Gambia government/MRCG at LSHTM; CVD-Mali, Bamako, Mali; and the KEMRI, Siaya County, Kenya.

## RESULTS

Saliva collection started in June, August, and November 2016 at the sites in Mali, Kenya, and The Gambia, respectively. During the ensuing 36 months at each site, saliva samples were collected from a total of 2082 cases with MSD and 2957 matched healthy controls. After excluding cases and controls with no documentation of rotavirus vaccine, rotavirus-negative cases with MSD, and cases with no rotavirus ELISA results, 224 rotavirus-positive cases with MSD and 307 matched controls were identified for saliva testing. HBGA phenotype results were obtained for 218 rotavirus-positive cases with MSD and 297 matched controls, with some results unavailable owing to inadequate saliva volume. In the subset of 42 saliva samples with genetic sequencing results, almost all samples aligned with the phenotypic results. Two samples showing likely contamination originally defined as secretors using ELISA were reassigned as nonsecretors after genotyping.

In the present analysis, 39% (n = 85), 25% (n = 54), and 36% (n = 79) of rotavirus-positive cases with MSD were from The Gambia, Mali, and Kenya, respectively (Table 1). A similar distribution of controls from each country was observed. As expected in a matched design, cases and controls had a similar median age (11 months) and sex distribution. All sites had a low proportion of partially immunized children, with The Gambia showing the largest proportion of partially immunized children (8% in cases and 10% in controls). Moderate-to-severe stunting occurred more frequently in The Gambia and Kenya than in Mali. Only in Kenya was stunting more common in rotavirus-positive cases than in controls.

**Table 1.** Demographic Characteristics of Rotavirus-Positive Cases With Moderate-to-Severe Diarrhea and Matched Healthy Controls

Characteristic	Cases or Controls, No. (%)^a^							
	The Gambia		Mali		Kenya		All Sites	
	Cases (n = 85)	Controls (n = 129)	Cases (n = 54)	Controls (n = 64)	Cases (n = 79)	Controls (n = 104)	Cases (n = 218)	Controls (n = 297)
Age, median, mo	11	11	10	11	11	11	11	11
Sex								
ȃMale	44 (52)	70 (54)	29 (54)	30 (47)	44 (56)	65 (62)	117 (54)	164 (55)
ȃFemale	41 (48)	59 (46)	25 (46)	34 (53)	35 (44)	40 (38)	101 (46)	133 (45)
Rotavirus vaccine								
ȃPartially immunized	7 (8)	13 (10)	1 (2)	5 (8)	4 (5)	3 (3)	22 (10)	45 (15)
ȃFully immunized	78 (92)	116 (90)	53 (98)	59 (92)	75 (95)	101 (97)	196 (90)	252 (85)
Stunting								
ȃNone or mild	71 (82)	106 (82)	47 (87)	56 (88)	58 (73)	84 (81)	174 (80)	246 (83)
ȃModerate to severe	16 (18)	23 (18)	7 (13)	8 (12)	21 (27)	20 (19)	44 (20)	51 (17)

Data represent no. (%) of cases or controls unless otherwise specified.

Table 2 shows the distribution of HBGA phenotypes at each site and across all sites among rotavirus-positive cases with MSD and cases. Across all sites, about 7% of cases and 20% of controls were defined as nonsecretors. This pattern was similarly observed in The Gambia and Kenya, while Mali had a lower prevalence of nonsecretors among rotavirus-positive cases with MSD (2%) than The Gambia and Kenya. Across all sites, 20% of cases and 36% of controls were defined as Lewis negative, and this pattern was consistent within each site.

**Table 2.** Secretor Status and Lewis Phenotypes in Rotavirus-Positive Cases With Moderate-to-Severe Diarrhea and Matched Healthy Controls

HBGA Phenotype	Cases or Controls, No. (%)							
	The Gambia		Mali		Kenya		All Sites	
	Cases (n = 85)	Controls (n = 129)	Cases (n = 54)	Controls (n = 64)	Cases (n = 79)	Controls (n = 104)	Cases (n = 218)	Controls (n = 297)
Secretor								
ȃNon-Secretor	8 (9.4)	26 (20.2)	1 (1.9)	13 (20.3)	6 (7.6)	21 (20.2)	15 (6.9)	60 (20.2)
ȃSecretor	77 (90.6)	103 (79.8)	53 (98.2)	51 (79.7)	73 (92.4)	83 (79.8)	203 (93.1)	237 (79.8)
Lewis status								
ȃLewis-negative	14 (16.5)	43 (33.3)	12 (22.2)	23 (35.9)	17 (21.5)	42 (40.4)	43 (19.7)	108 (36.4)
ȃLewis-positive	71 (83.5)	86 (66.7)	42 (77.8)	41 (64.1)	62 (78.5)	62 (59.6)	175 (80.3)	189 (63.6)

Abbreviation: HBGA, histo-blood group antigen.

We examined the crude association between secretor status and rotavirus vaccine failure and found that nonsecretors were 70% less likely than secretors to be rotavirus-positive case with MSD (matched odds ratio [mOR, 0.30 [95% CI: 0.16–0.56]) (Table 3). After excluding stunted children, partially vaccinated children, and both types together, the odds of being a rotavirus-positive case with MSD did not change meaningfully (mOR, 0.25 [95% CI: 0.10–0.56]), compared with the crude estimate. Examination of the crude association between Lewis status and rotavirus vaccine failure demonstrated that Lewis-negative children were 61% less likely than Lewis-positive children to be a rotavirus-positive case with MSD (mOR, 0.39 [95% CI: 0.25–0.62]). After excluding stunted children, partially vaccinated children, and both types together, the odds of being a rotavirus-positive case with MSD did not change meaningfully (mOR, 0.29 [95% CI: 0.16–0.55]), compared with the crude estimate.

**Table 3.** Associations Between Lewis and Secretor Status and Rotavirus Vaccine Failure

HBGA Phenotype	mOR (95% CI)			
	Unadjusted	Exclusion of Stunted Children	Exclusion of Partially Vaccinated Children	Exclusion of Both Stunted and Partially Vaccinated Children
Secretor				
ȃSecretor	Reference	Reference	Reference	Reference
ȃNon-Secretor	0.30 (0.16–0.56)	0.31 (0.14–0.64)	0.27 (0.14–0.52)	0.25 (0.10–0.56)
Lewis status				
ȃLewis-positive	Reference	Reference	Reference	Reference
ȃLewis-negative	0.39 (0.25–.62)	0.32 (0.18–0.57)	0.37 (0.23–0.61)	0.29 (0.16–0.55)

Abbreviations: CI, confidence interval; HBGA, histo-blood group antigen; mOR, matched odds ratio.

**Figure 1.**
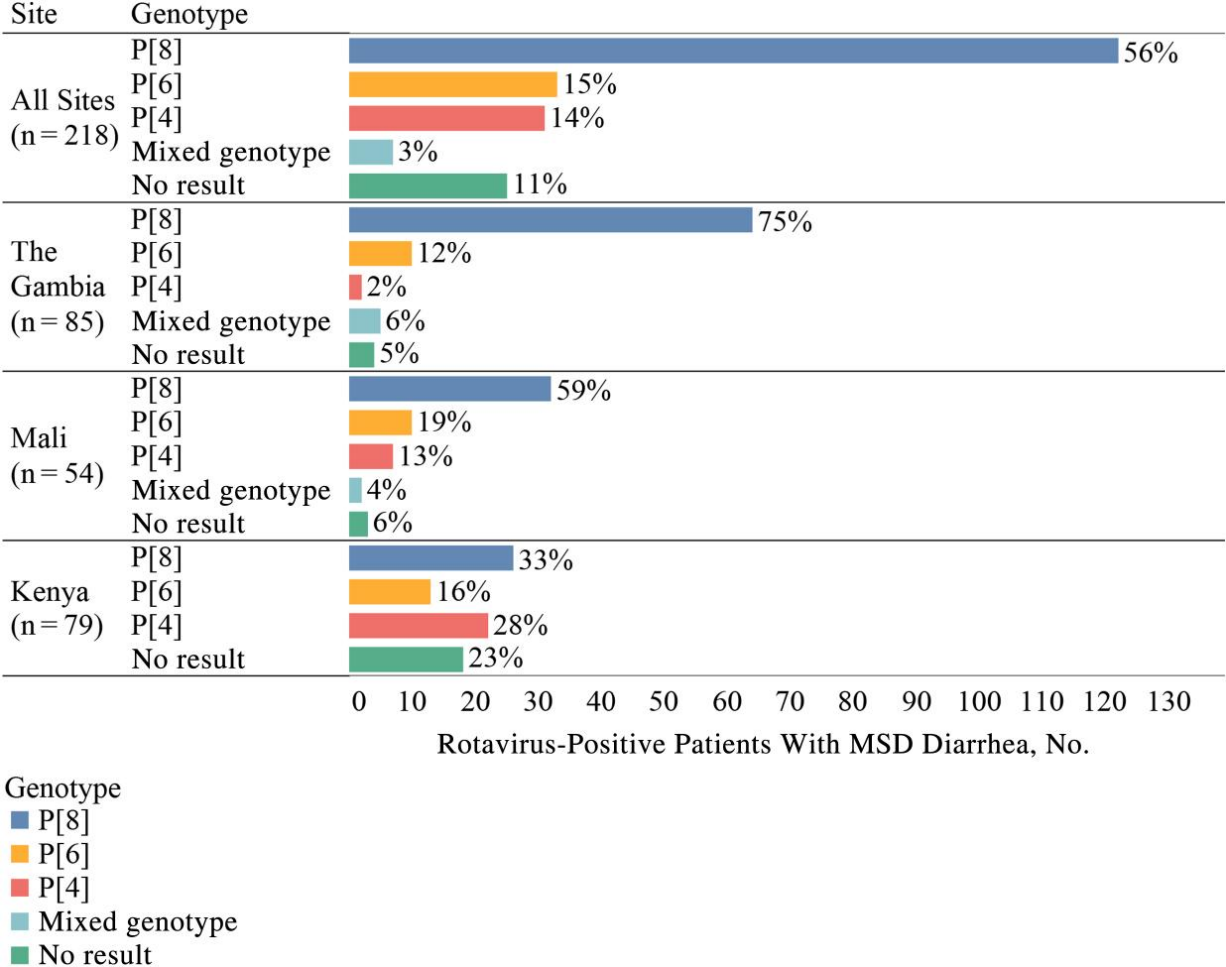
Rotavirus genotype distribution among rotavirus-positive case with moderate-to-severe (MSD) diarrhea, overall and by site.

Rotavirus genotyping results were available for 193 of the 218 rotavirus-positive cases with MSD (88%) (Figure 1). Across all sites and within each site, P[8] was the most common infecting genotype. P[6] was the second most common infecting genotype for all sites except for Kenya, where P[4] was more common.

HBGA phenotypes were further compared (across all sites) among cases and matched controls within each infecting genotype (Table 4). Among P[8] rotavirus–infected cases with MSD and their matched controls, nonsecretors were 81% less likely than secretors to be a rotavirus-positive case (mOR, 0.19 [95% CI: 0.06–0.54]). Among P[6] rotavirus–infected cases with MSD and their matched controls the relationship between secretor status and rotavirus vaccine failure was not significant (mOR, 1.23 [95% CI: 0.38–3.92]). Nonsecretor status absolutely protected against infection with the P[4] strain. Among P[8] rotavirus–infected cases with MSD and their matched controls, Lewis-negative children were 81% less likely than Lewis-positive children to be a case (mOR, 0.19 [95% CI: 0.08–0.42]). Among P[6] rotavirus–infected cases with MSD and their matched controls the relationship between Lewis status and rotavirus vaccine failure was not statistically significant (mOR, 4.2 [95% CI: 0.88–19.64]). Among P[4] rotavirus–infected cases with MSD and their matched controls, Lewis-negative children were 94% less likely than Lewis-positive children to be cases (mOR, 0.06 [95% CI: 0.01–0.48]).

**Table 4.** Associations Between Lewis and Secretor Status and Rotavirus Vaccine Failure, by Genotype

HBGA Phenotype	P[8]			P[6]			P[4]		
	Rotavirus-Positive Cases, No. (%)	Controls, No. (%)	mOR (95% CI)	Rotavirus-Positive Cases, No. (%)	Controls, No. (%)	mOR (95% CI)	Rotavirus-Positive Cases, No. (%)	Controls, No. (%)	mOR (95% CI)
Secretor Status									
ȃSecretor	118 (97)	137 (82)	Reference	26 (79)	33 (81)	Reference	32 (100)	28 (78)	Reference
ȃNon-Secretor	4 (3)	31 (18)	0.19 (0.06-0.54)	7 (21)	8 (19)	1.23 (0.38-3.92)	0 (0)	8 (22)	*
Lewis status									
ȃLewis-positive	113 (93)	118 (70)	Reference	10 (30)	19 (46)	Reference	31 (97)	19 (53)	Reference
ȃLewis-negative	9 (7)	50 (30)	0.19 (0.08–0.42)	23 (70)	22 (54)	4.2 (0.88–19.64)	1 (3)	17 (47)	0.06 (.01–.48)

Abbreviations: CI, confidence interval; HBGA, histo-blood group antigen; mOR, matched odds ratio. *Absolute protection.

## DISCUSSION

This is one of the first studies assessing the relationship between secretor and Lewis phenotypes and clinical rotavirus vaccine failure in sub-Saharan Africa. We initially hypothesized that null HBGA phenotypes would be associated with increased clinical rotavirus vaccine due to reduced vaccine take compounded by continued susceptibility to P[6] infections. While we found no significant association between null HBGA phenotypes and vaccine failure among P[6] rotavirus–infected cases with MSD and their matched controls, the mOR point estimate for Lewis-negative individuals was >4, even in a small sample.

As expected, given their natural immunity, individuals with P[8] infections were significantly less likely to be nonsecretors or Lewis negative. Given that 58% of rotavirus vaccine failures occurred in patients with P[8] infections, we observed that null HBGA phenotypes were associated with a reduced risk of clinical rotavirus vaccine failure overall. The protective effect for P[8] and P[4], which combined are the majority of infections in the current study, offset any potential increased risk for P[6] infections. This relationship persisted after exclusion of children with other factors that would independently contribute toward rotavirus vaccine failure, such as those who were stunted or partially immunized.

The hypothesis that HBGA phenotype interacts with rotavirus vaccine and influences the subsequent immune response has been studied in several settings with varying results. In Malawi there was no significant difference in Rotarix vaccine virus shedding or seroconversion between secretors and nonsecretors [24]. In Nicaragua, no difference was observed in seroconversion rates between secretors and nonsecretors for Rotarix or RotaTeq [27]. Alternatively, in Ghana, 41% of secretors seroconverted to Rotarix, compared with 13% of nonsecretors (relative risk, 3.2 [95% CI: 1.2–8.1]) [25] but the investigators found no association for Lewis phenotype and seroconversion. In Pakistan, blood group O secretors were 2.8 (95% CI: 1.5–5.2) times more likely to seroconvert to Rotarix compared with nonsecretors and 1.7 (1.1–2.7) times more likely compared with non–blood group O secretors [23]. No association was observed for Lewis phenotype.

Only two studies have examined the association between HBGA phenotypes and rotavirus vaccine failure in low- and middle-income settings. In a case-control study among vaccinated children in Malawi, 28% of controls were nonsecretors, and 26% were Lewis negative [24], compared with 20% and 36%, respectively, of controls in our study. In Malawi, nonsecretors had a 61% reduced risk of rotavirus vaccine failure (odds ratio, 0.39 [95% CI: 0.20–0.75]) compared with community controls, which was similar to our study results (mOR, 0.30 [0.16–0.56]). Both studies demonstrated a decreased risk of rotavirus vaccine failure when the infecting rotavirus strain among case patients was a P[8] or P[4] genotype in nonsecretors compared with secretors, and no association in those with P[6] rotavirus.

Both studies found similar genotype-specific associations with Lewis status and both P[8] and P[4] infections with a decreased risk of rotavirus vaccine failure in Lewis-negative participants, compared with those who were Lewis positive. In Malawi, among patients with P[6] rotavirus infection, Lewis-negative participants were at a significantly increased risk of rotavirus vaccine failure, compared with Lewis-positive participants (odds ratio, 3.2 [95% CI: 1.4–7.2]). With P[6] infections contributing 28% of rotavirus cases in Malawi, the association between Lewis phenotype and rotavirus vaccine failure overall was not significant, likely owing to equally opposing effect from P[8]/P[4] and P[6] infections. In our study, among cases with P[6] rotavirus infection, the direction and magnitude of the mOR point estimate was similar to those in Malawi, but results were not significant. With P[6] infections contributing only 15% of rotavirus cases in our study, we found a statistically significant 61% reduced risk of rotavirus vaccine failure in Lewis-negative, compared with Lewis-positive participants. The decreased risk for P[8]/P[4] infections offset any potential increased risk for P[6] infections.

Results of a study within a randomized controlled trial in Bangladesh were different from both our findings and those of the study in Malawi. In Bangladesh, only 15% of controls were defined as Lewis negative [22]. The association between null HBGA phenotypes and rotavirus diarrhea was examined in both vaccinated and unvaccinated groups. Among unvaccinated children, nonsecretors had a significantly reduced risk of rotavirus diarrhea and were resistant to P[4] infections, with no difference in P[8] or P[6] infections. Among vaccinated children, there was no association between secretor or Lewis phenotype and rotavirus diarrhea. Differences in immunogenicity and clinical rotavirus vaccine failure in studies that have explored associations with HBGA phenotype are likely due to variability in the severity of rotavirus diarrhea, distributions of both infecting rotavirus genotype and HBGA phenotype, and inconsistent methods used to define null HBGA phenotypes across studies.

Our study had several limitations. First, HBGA phenotyping was determined by ELISA, with optical density cutoff values based on regression analysis of a standard curve. There are various methods to determine optical density cutoffs to determine positivity for each HBGA antigen, and these tend to differ across studies. We conducted genotyping on a subset of samples, with almost 100% consistency between phenotype and genotype, which supports our method. Second, two methods to identify rotavirus genotype were used in this study. There was no opportunity to compare the results obtained by method, and this may have introduced bias in the genotyping results, though we have no evidence of that. The genotype distribution was similar for both methods (60%–70% P[8], 20%–25% P[4]). Third, while we conducted sensitivity studies to account for other variables associated with rotavirus vaccine failure, our case-control study design did not allow us to account for other important risk factors, such as enteric burden and maternal rotavirus antibodies, at vaccination. Differences in these factors between patients and controls could explain some of the relationships identified.

In summary, our study supported other analyses showing a statistically significant relationship between null HBGA phenotypes and decreased rotavirus vaccine failure in a population with P[8] as the most common infecting genotype. While we did not find a significant relationship between Lewis status and increased risk of rotavirus vaccine failure for P[6] infections, further studies are needed in populations with both an increased prevalence of null HBGA phenotypes and burden of P[6] rotavirus diarrhea to clarify the role of host genetics in reduced rotavirus vaccine effectiveness.

## Supplementary Data

Supplementary materials are available at *Clinical Infectious Diseases* online. Consisting of data provided by the authors to benefit the reader, the posted materials are not copyedited and are the sole responsibility of the authors, so questions or comments should be addressed to the corresponding author.

## Supplementary Material

ciac910_Supplementary_Data

## Notes

***Acknowledgments.*** We express our deep gratitude to the families who participated in these studies, the clinical and field staff for their exceptional hard work and dedication, and the physicians, administrators, and health officials at every site who generously provided facilities and support for the conduct of the study. We are grateful to Catherine Johnson, Chris Focht, and Nora Watson at the Emmes Company for expert data management and reporting. Special thanks go to Carl Kirkwood, Duncan Steele, and Anita Zaidi at the Bill & Melinda Gates Foundation for helpful oversight and suggestions and to the following members of our International Scientific Advisory Committee for providing insightful comments and guidance: Janet Wittes (chair), George Armah, John Clemens, Christopher Duggan, Stephane Helleringer, Ali Mokdad, James Nataro, and Halvor Sommerfelt. We would also like to acknowledge Jennifer Jones, Joseph Nkeze, and Deanna Toema from the Center for Vaccine Development and Global Health, who assisted with coordination of shipments and rotavirus genotyping.

***Disclaimer*.** The findings and conclusions contained within are those of the authors and do not necessarily reflect positions or policies of the Bill & Melinda Gates Foundation, the National Institutes of Health, the Centers for Disease Control and Prevention, or any of the collaborating partners in this project. Funders of the study had no role in study design, data collection, data analysis, data interpretation, writing of the report, or the decision to submit the article for publication. Names of specific vendors, manufacturers, or products are included for public health and informational purposes; inclusion does not imply endorsement of the vendors, manufacturers, or products by the Centers for Disease Control and Prevention or the US Department of Health and Human Services.

***Financial support*.** This study was funded by the Bill & Melinda Gates Foundation (grants OPP1116751 and OPP1111236), and the National Institute of Allergy and Infectious Diseases, National Institutes of Health (grant F31AI126629 to L. M. S. and R37 AI032042 to M. E. H.).

***Supplement sponsorship*.** This supplement was sponsored by the Bill & Melinda Gates Foundation. This study is based on research funded in part by grants from the Bill & Melinda Gates Foundation (OPP1111236/OPP1116751).
