# Supplementary material for: Histo-Blood Group Antigen Null Phenotypes Associated With a Decreased Risk of Clinical Rotavirus Vaccine Failure Among Children <2 Years of Age Participating in the Vaccine Impact on Diarrhea in Africa (VIDA) Study in Kenya, Mali, and the Gambia
Source: Clin Infect Dis. 2023 Apr 19;76(Suppl 1):S153–61. doi: 10.1093/cid/ciac910 (PMC10116560; doi:10.1093/cid/ciac910)
Supplement: ciac910_Supplementary_Data [file ciac910_supplementary_data.docx]

**Supplementary Methods**

**Detection of Histo-blood Group Antigens and Determination of Secretor Status in Saliva**

The AB histo-blood group and Lewis a, Lewis b phenotypes were determined in 537 pediatric saliva samples by ELISA. Briefly, ELISA plates (Nunc MaxiSorp flat-bottom plates) were coated with saliva diluted 1:1000 in PBS, pH 7.4, incubated overnight at 4°C. Plates were washed with PBS-Tween (0.05%) and blocked with 5% NFDM in PBS for 1hr at 37°C. Plates used for determination of HGBAs were washed and incubated for 1hr at 37°C with mouse monoclonal antibodies anti-BG-2 (Type A, Biolegend #921902), anti-BG-3 (Type B, Biolegend #92202), anti-BG-5 (Lewis a, Biolegend #922202), anti-BG-6 (Lewis b, Biolegend #922302) diluted 1:300 in 1% NFDM in PBS, followed by washing and incubation for 1hr at 37°C with anti-mouse IgG-HRP [(KPL # 474-1802) for anti-BG-2 and anti-BG-5] or anti-mouse IgM-HRP [(KPL# 074-1803) for anti-BG-3 and anti-BG-6] antibodies diluted at 1:1000 and 1:5000, respectively, in 1% NFDM in PBS. Plates used for determination of secretor status were washed following block and incubated with UEA-1 (*Ulex europaeus* agglutinin I)-Peroxidase 0.5mg/ml (Sigma # L8146) diluted 1:1600 in 1% NFDM in PBS for 1hr at 37°C. Both sets of plates were washed and developed with TMB Microwell Peroxidase Substrate System (KPL #50-76-00). Development was stopped with 1M phosphoric acid, plates were read on Thermo Labsystems Multiskan FC™ microplate reader. OD values were compared to cutoff values calculated for each antigen based on bend-point determination of 4PL regression analysis of a standard curve.

**Determination of Cutoff Values**

In order to determine the most appropriate cutoff values for our ELISA results, we generated dilution curves for a positive control sample for each of the blood type antigens tested. The data from the dilution series was fitted with a four-parameter logistic (4PL) regression curve of the form:


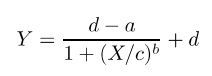


The bend points on the curve are the points at which the slope of the linear portion of the curve changes as it approaches the lower or upper asymptote. The bend point values were determined by taking the mixed partial derivative with respect to the slope (*b*) and *X*:


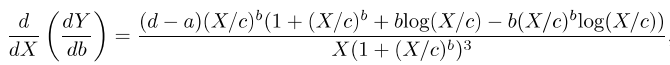


By setting this equation to zero and solving for *X*, the point at which the slope is zero, the bend point, is derived [1]. We did this by entering the dilution factors and resulting OD values into a program in R. We used the lower bend point as the cutoff point for each assay.

| Cutoff Points based on lower bend points: | **Type A** | **Lewis^a^** | **Type B** | **Lewis^b^** | **UEA-1** |
| --- | --- | --- | --- | --- | --- |
| mAb 1:300 | 0.297 | 0.0907 | 0.254 | 0.316 | 0.205 |

**Isolation of gDNA and SNP Analysis in Saliva**

Genomic DNA was isolated from saliva cell pellets using the QIAamp DNA Blood Mini Kit (Qiagen #51104). PCR amplification of the FUT2 gene from the isolated gDNA was performed using Phusion High -Fidelity DNA Polymerase (NEB #M0530S) and the forward primer: 5’-ACACACCCACACTATGCCTGCAC-3’ and reverse primer: 5’-ACTTGCAGCCCAACGCATCTT-3’[2]. The 50µl reaction mixture contained 20µl DNA, 10µl 5X Phusion HF buffer, 200µM dNTPs, 0.5µM of each primer, 3% DMSO, and 1 unit of Phusion® High-Fidelity DNA polymerase. The PCR program was 98°C for 30s, 35 cycles of 98°C for 5min, 72°C for 50s, followed by 72°C for 7min, yielding a 1.2kb product. The PCR product was cleaned using QIAquick PCR purification Kit (Qiagen #28104) and submitted for sequencing using forward and reverse primers (see Table below).

| Primer | Sequence 5’-3’ | Reference |
| --- | --- | --- |
| ForSeq1 | CCA GCT AAC GTG TCC CGT TTT CC | [2] |
| RevSeq1 | TGC CTC CCT CAA GAT GAG TGC C | [2] |
| RevSeq2 | CTG TCC CCC TTA CTC AAG CAC TAA | This study |
| ForSeq2 | TGG ACC TTC TAC CAC CAC CTC | This study |
| RevSeq3 | AAT TGG CCA GGT AGA TGG TGT C | This study |

FUT2 SNPs selected to determine secretor/ non-secretor genotype were chosen based on the following information:

1. Geographical distribution of haplotypes as described in Ferrer-Admetlla et al., 2009 [2]: ubiquitous haplotypes H3 and H10; H7- the haplotype carrying the se^428^ null allele, known to be present in nearly half of the African population; H17 – haplotype exclusive to Sub-Saharan Africa (SSAFR). Additional SNPs reported as not being present in the SSAFR population were included as negative controls.
2. All Null alleles of the FUT2 gene conferring nonsecretor status reported by Scharberg et al. 2016 [3].

**Rotavirus genotyping**

The majority of rotavirus-positive specimens from Mali were genotyped to determine G- and P- genotypes according to the following methodology.

**Stool processing and nucleic acid extraction for specimens from Mali only**

Stool specimens were diluted to 10% (w/v or v/v) suspensions in Vertrel XF (Miller Stephenson) and centrifuged at 1000 x g for 10 minutes. RNA was isolated using a QIAamp Viral RNA Mini kit (Qiagen) according to the manufacturer’s instructions. No internal RNA extraction controls were used. If RT-PCR for VP7 and VP4 genes failed, RNA extraction and RT-PCR were repeated and performed up to three times to ensure that a product would be produced for genotype determination (3 failures out of 138 specimens positive for rotavirus by ELISA).

**Rotavirus VP7 and VP4 genotyping for specimens from Mali only**

VP7 and VP4 genes were amplified from purified RNA using the One-step RT-PCR kit (Qiagen). VP7 was amplified using forward primer “ddrv1” (5' GCT CYT TTT RAT GTA TGG TAT TGA ATA TAC CAC 3’) and reverse primer “ddrv2" (5' CTT TAA AAT ANA YDG ADC CWR TYG GCC A 3’). VP4 was amplified using forward primer "con3" (5' TGG CTT CGC TCA TTT ATA GAC A 3’) and reverse primer "con2d" (5' ATT TCG GAC CAT TTA TAD CC 3'). PCR products were electrophoresed on a 1.5% agarose gel and once amplicons were observed, the remaining PCR product was purified using a QIAquick PCR purification kit (Qiagen). Purified DNA from the VP7 PCR product was sequenced using VP7 forward primer (5' YTT TTR ATG TAT GGT ATT GAA TAT ACC AC 3’) and VP7 reverse primer (5' AAA ATA NAY DGA DCC WRT YGG CCA 3'). Purified DNA from the VP4 PCR product was sequenced using VP4 forward primer (5' TGG CTT CGC TCA TTT ATA GAC A 3’) and VP4 reverse primer (5' ATT TCG GAC CAT TTA TAD CC 3’). G- and P-genotypes were determined by performing BLAST analyses.

All rotavirus-positive specimens from Kenya, The Gambia and a subset of samples from Mali were genotyped according to the following methodology. The subset of sample from Mali were genotyped using a genotype agnostic approach whereby VP4 and VP7 genes were amplified by RT-PCR and then sequenced to determine the genotype. The remaining samples were genotyped by using multiplexed RT-PCR as well as sequencing for a subset of samples. The differences in these methodologies are unlikely to have an effect on genotype determination.

**Stool processing and nucleic acid extraction for all remaining specimens**

A 10% stool suspension was prepared for each sample using phosphate-buffered saline and RNA was extracted from the suspension using either the MagMAX™-96 Viral RNA Isolation Kit (ThermoFisher Scientific, Vilnius, Lithuania) on the KingFisher™ Flex Purification System (ThermoFisher Scientific, Vantaa, Finland) or the MagNA Pure Compact RNA extraction kit on the MagNA Pure Compact instrument (Roche Applied Science, Indianapolis, IN, USA) following the manufacturer's instructions. Prior to each of the above extraction procedures, 2 µL of 109 unit/ µL of MS2 bacteriophage RNA (ZeptoMetrix, Buffalo, NY, USA), were spiked into a 48 µL or 98 µL volume of 10% stool suspension to serve as process internal controls.

**Rotavirus VP7 and VP4 genotyping for all remaining specimens**

Genotyping was performed using reverse-transcription polymerase chain reaction (RT-PCR) to determine the G and P-genotypes. VP7 and VP4 genotyping RT-PCR was performed using a conventional multiplexed one-step amplification process with slight modifications [4]. In brief, the genotype G2 (G2-R4), G4 (G4-R2) and G9 (G9-R2) specific primers were replaced with an updated versions G2 (G2-R1: TAT GTA GTC CAT YGT ATT AGT), G4 (G4-R1: GAG CAT TCG MTA ATA MTG ATA ATA C), and G9 (G9-R3: CAG AGT ATY YTT CCA TTC HGT ATC TCC) primers. The VP7 and VP4 conventional multiplexed one-step RT-PCR genotyping product was electrophoresed on 3% agarose gels containing GelRed (Biotium, Heyward, CA, USA) for 2-3hrs at 100 V and products were detected under UV transillumination or were analyzed on the LabChip®GX instrument (Caliper, Life Sciences, MA, USA) using the HT DNA 1K or 5K reagent kit (Dual protocol DNA Analysis and Quantitation) with the HT Extended Range LabChip (Caliper Life Sciences, MA, USA) as described previously [4]. Non-typeables samples and a selection of common and less common genotypes were sequenced using Sanger and next generation sequencing methods as described previously [5,6].

**Results of gDNA Isolation FUT2 SNP Analysis in Saliva**

| **Observed Phenotype** | **n** | **Genotype** | **Note** |
| --- | --- | --- | --- |
| Nonsecretor | 21 | se^171,216,428,739,960^ |  |
| Nonsecretor | 1 | se^428^ | Unable to determine status of other SNPs due to quality of sample. |
| Nonsecretor | 1 | se^(171),(216),(357),(428),739,778^ |  |
| Nonsecretor | 1 | se^(171),(216),(357),(428),(739),778,960^ |  |
| Secretor | 7 | se^(171),(216),(357),(428),(739),(960)^ |  |
| Secretor | 1 | se^(216),(357),(481),(960)^ |  |
| Secretor | 1 | se^(216),(481),(960)^ |  |
| Secretor | 2 | se^(171),(216),(428),(481),(739),(960)^ |  |
| Secretor | 1 | se^(171),(216),(428),(481),(739)^ | Status of 960 unknown in this subject |
| Secretor | 1 | se^(357),(481),(960)^ |  |
| Secretor | 2 | se^(216),357^ | Status of 960 unknown in these subjects |
| Secretor | 1 | se^357^ |  |
| Secretor | 1 | se^(357)^ |  |
| Secretor | 1 | se^(960)^ |  |
| Also checked SNPs at 244, 302, 385, 400, 412, 429, 443, 543, 569, 571, 628, 658, 664,685, 688, 760, 818, 849, 868 all HmWT for all samples. | | | |

[1] Sebaugh JL, McCray PD. Defining the linear portion of a sigmoid-shaped curve: bend points. Pharm Stat 2003;2:167–74. doi:10.1002/pst.62.

[2] Ferrer-Admetlla A, Sikora M, Laayouni H, Esteve A, Roubinet F, Blancher A, et al. A Natural History of FUT2 Polymorphism in Humans. Mol Biol Evol 2009;26:1993–2003. doi:10.1093/molbev/msp108.

[3] Scharberg EA, Olsen C, Bugert P. The H blood group system. Immunohematology 2016.

[4] Esona MD, Gautam R, Tam KI, Williams A, Mijatovic-Rustempasic S, Bowen MD. Multiplexed one-step RT-PCR VP7 and VP4 genotyping assays for rotaviruses using updated primers. J Virol Methods 2015;223:96–104. doi:10.1016/j.jviromet.2015.07.012.

[5] Esona MD, Geyer A, Page N, Trabelsi A, Fodha I, Aminu M, et al. Genomic characterization of human rotavirus G8 strains from the African rotavirus network: relationship to animal rotaviruses. J Med Virol 2009;81:937–51. doi:10.1002/jmv.21468.

[6] Katz EM, Esona MD, Betrapally NS, De La Cruz De Leon LA, Neira YR, Rey GJ, et al. Whole-gene analysis of inter-genogroup reassortant rotaviruses from the Dominican Republic: Emergence of equine-like G3 strains and evidence of their reassortment with locally-circulating strains. Virology 2019;534:114–31. doi:10.1016/j.virol.2019.06.007.
